# Scientific Items

**Published:** 1878-09-20

**Authors:** 


### *Scientific Items.*

### BRUTE CEREMONIES.

Proof that the modifications of conduct called "manners" and "behavior" arise long before those which political and religious restraints cause, is yielded by the fact that, besides preceding social evolution, they precede human evolution: they are traceable among the higher animals. The dog afraid of being beaten, comes crawling up to his master, clearly manifesting the desire to submit. Nor is it solely to human beings that dogs use such propitiatory actions: they do the like one to another. All have occasionally seen how, on the approach of some formidable-looking Newfoundland or mastiff, a small spaniel, in the extremity of its terror, throws itself on its back with legs in the air. Instead of threatening resistance by growls and showing of teeth, as it might have done had not resistance been hopeless, it spontaneously assumes the attitude that would result from defeat in battle, tacitly saying, "I am conquered, and at your mercy." Clearly, then, besides certain modes of behavior expressing affection, which are established still earlier in creatures lower than man, there are established certain modes of behavior expressing subjection.—HERBERT SPENCER, *in Popular Science Monthly for February.*

### VELOCITY OF NERVE-IMPULSES.

The earliest experiments were made with reference to the rapidity of movement through the nerves. The first attempt to measure the velocity of nervous impulses proceeding from the brain under action of the will was made long ago by Haller. He ascertained, by reading aloud with great rapidity extracts from the "Æneid," the average number of letters which he could pronounce in one minute. Then he calculated the length of the nerve from the brain to the muscles of the tongue and mouth. Each letter he regarded as requiring a nervous impulse. He was obliged then only to multiply the number of letters spoken in each minute by the length of the nerve. This gave as a result that the rate of nervous transmission from the brain was about 150 feet a second.

### HATCHING SILKWORMS AT WILL.

According to *Galignani,* M. Duclaux, professor of sciences at Lyons, has discovered a method of hatching silkworm eggs at will. If, when the eggs are not more than two or three days old, they are rubbed with a brush, subjected to the action of electricity, dipped for half a minute in concentrated sulphuric acid, or chlorhydric, nitric, acetic, or tartaric acids, they will hatch out, and as the mulberry is in full leaf two crops of worms are thus obtained in one season. Immersion for a few seconds in water heated to 122°F. is said to be equally efficacious. The worms from the artificial hatching are said to be hardier than those reared in the ordinary manner.

### THE NEW METAL GALLIUM.

Sufficient quantities of this of this new element have now been obtained to make us better acquainted with its physical and chemical properties. It is found to be a hard and malleable metal; it takes under the hammer the polish of the anvil, but rapidly grows harder and brittle, and is then liable to fly to pieces. In spite of its relatively considerable hardness, gallium leaves on paper strongly-defined marks of a bluish-gray color. It retains its lustre in the atmosphere of a labatory constantly loaded with acid vapors, and undergoes no alteration in appearance in boiled water. In aerated water it tarnishes slightly.—*Jour. Phar.*

